# Second primary neoplasms among 53 159 haematolymphoproliferative malignancy patients in Sweden, 1958–1996: a search for common mechanisms

**DOI:** 10.1054/bjoc.2001.1998

**Published:** 2001-09

**Authors:** C Dong, K Hemminki

**Affiliations:** Department of Biosciences at Novum, Karolinska Institute, Huddinge, 141 57, Sweden

**Keywords:** second malignancies, haematolymphoproliferative disorders, Epstein–Barr virus, immunosuppression, follow-up study

## Abstract

The Swedish Family-Cancer Database was used to analyse site-specific risk of second primary malignancies following 53 159 haematolymphoproliferative disorders (HLPD) diagnosed between 1958 and 1996. Standardized incidence ratio (SIR) of a second malignancy was calculated as the ratio of observed to expected numbers of second malignancies by applying site-, sex-, age-, period-, residence- and occupation-specific rates in the corresponding population in the Database to the appropriate person-years at risk. Among 18 960 patients with non-Hodgkin's lymphoma (NHL), there was over a 3-fold significant increase in cancer of the tongue, small intestine, nose, kidney and nervous system, squamous cell carcinoma (SCC) of the skin, NHL, Hodgkin's disease (HD) and lymphoid and myeloid leukaemia. Among 5353 patients with HD, there was over a 4-fold significant increase in cancer of the salivary glands, nasopharynx and thyroid, NHL and myeloid leukaemia, and over a 1.6-fold increase in cancer of the stomach, colon, lung, breast, skin (melanoma and SCC), nervous system and soft tissues and lymphoid leukaemia. Among 28 846 patients with myeloma and leukaemia, there was a significant increase in cancer of the skin, nervous system and non-thyroid endocrine glands and all HLPD except for myeloma. Our findings showed some clustering between first and second primaries among Epstein–Barr virus-, ultraviolet radiation- and immunosuppression-related cancers. © 2001 Cancer Research Campaignhttp://www.bjcancer.com


*British Journal of Cancer* (2001) **85**(7), 997–1005
© 2001 Cancer Research Campaign
doi: 10.1054/ bjoc.2001.1998, available online at http://www.idealibrary.com on IDEAL

http://www.bjcancer.com

### Second primary neoplasms among 53 159 haematolymphoproliferative malignancy patients in Sweden, 1958–1996: a search for common mechanisms

**C Dong and K Hemminki**

Department of Biosciences at Novum, Karolinska Institute, 141 57 Huddinge, Sweden

**Summary** The Swedish Family-Cancer Database was used to analyse site-specific risk of second primary malignancies following 53 159 haematolymphoproliferative disorders (HLPD) diagnosed between 1958 and 1996. Standardized incidence ratio (SIR) of a second malignancy was calculated as the ratio of observed to expected numbers of second malignancies by applying site-, sex-, age-, period-, residence- and occupation-specific rates in the corresponding population in the Database to the appropriate person-years at risk. Among 18 960 patients with non-Hodgkin's lymphoma (NHL), there was over a 3-fold significant increase in cancer of the tongue, small intestine, nose, kidney and nervous system, squamous cell carcinoma (SCC) of the skin, NHL, Hodgkin's disease (HD) and lymphoid and myeloid leukaemia. Among 5353 patients with HD, there was over a 4-fold significant increase in cancer of the salivary glands, nasopharynx and thyroid, NHL and myeloid leukaemia, and over a 1.6-fold increase in cancer of the stomach, colon, lung, breast, skin (melanoma and SCC), nervous system and soft tissues and lymphoid leukaemia. Among 28 846 patients with myeloma and leukaemia, there was a significant increase in cancer of the skin, nervous system and non-thyroid endocrine glands and all HLPD except for myeloma. Our findings showed some clustering between first and second primaries among Epstein–Barr virus-, ultraviolet radiation- and immunosuppression-related cancers. © 2001 Cancer Research Campaign http://www.bjcancer.com

**Keywords**: second malignancies; haematolymphoproliferative disorders; Epstein–Barr virus; immunosuppression; follow-up study

The haematolymphoproliferative disorders (HLPD) are a heterogeneous group of neoplasms accounting for some 8.6% of all tumours in males and 7.1% of all tumours in females reported to the Swedish Cancer Registry in 1996 (Centre for Epidemiology, 1999). Non-Hodgkin's lymphoma (NHL) is the most common subgroup and its age-standardized incidence has increased annually by 2.5% on average since 1977, while the corresponding rates of Hodgkin's disease (HD), multiple myeloma ('myeloma') and leukaemia have been relatively stable in Sweden. The rapid increase in NHL incidence and mortality is a worldwide trend (Groves et al, 2000). Risk factors for HLPD are generally poorly understood (Adami et al, 1995; Groves et al, 2000). Immunodeficiency states, both genetic and those induced by medications or illness, are associated with a markedly elevated risk of NHL (Kinlen, 1992; Adami et al, 1995; Groves et al, 2000). There is evidence that viruses, particularly the Epstein–Barr virus (EBV), may play a role in the aetiology of NHL and HD (IARC, 1996, 1997). Chemotherapeutic agents, pesticides and organic solvents are associated with NHL risk, while ionizing radiation is associated with myeloma and leukaemia (Groves et al, 2000). Recently, suggestive evidence has been reported that exposure to ultraviolet radiation (UVR) may increase the risk for NHL by causing immune impairment (Cartwright et al, 1994; Adami et al, 1995; Groves et al, 2000; IARC, 2001). A family history of HLPD is a risk factor in a small proportion of HLPDs (Horwitz, 1997; Hemminki et al, 1998; Yuille et al, 1998; Capalbo et al, 2000; Shugart et al, 2000; Wiernik et al, 2000).

The aetiology of second primary cancers is likely to be multifactorial and may include any of the above factors and interactions among these (Greene and Wilson, 1985; Storm and Prener, 1985; Travis et al, 1991; Adami et al, 1995; Sankila et al, 1995; Vaittinen and Hemminki, 2000; Dong and Hemminki, 2001a). Shared risk factors may cause certain malignancies to cluster more frequently than expected. The study of multiple primary malignancies following HLPD therefore has aetiological and clinical importance. In this nationwide study, we used the Swedish Family-Cancer Database to systematically analyse the site-specific risk of second primary neoplasm among 53 159 patients diagnosed with a HLPD during 1958–1996 in Sweden (Vaittinen and Hemminki, 2000; Dong and Hemminki, 2001a).

*Received 15 January 2001*
*Revised 7 June 2001*
*Accepted 8 June 2001*

*Correspondence to:* K Hemminki

### SUBJECTS AND METHODS

### The Swedish Family-Cancer Database

The Swedish Family-Cancer Database, updated in 1999, was formed from the Second Generation Register maintained by Statistics Sweden and linked by the individually unique national registration number to the Swedish Cancer Register at the National Board of Health and Welfare. The Database includes all persons born in Sweden after 1934 with their biological parents, totalling over 9.6 million individuals (Hemminki and Vaittinen, 1999; Hemminki and Dong, 2000a). Since 1958, all new cases of tumour in Sweden have been reported to the Swedish Cancer Registry. An almost 100% coverage has been achieved by compulsory reporting by clinicians who diagnose a neoplasm, while pathologists and cytologists must report separately any diagnosis of malignancy made on specimens they receive. The site of tumour is coded to a 4-digit diagnostic code based on the 7th revision of the International Classification of Diseases (ICD-7) by the Swedish Cancer Registry (see footnote to Table 1). We did not separate acute and chronic leukaemia because acute lymphoid is less than 30% of all lymphoid leukaemia, and because chronic myeloid is less that 20% of all myeloid leukaemia; furthermore, diagnostic criteria for leukaemia have been modified over the years. Basal cell carcinoma of the skin is not registered in the Cancer Registry. The stage of the disease at diagnosis or treatment is not recorded.

### Patients

All patients with an initial HLPD diagnosed between 1 January 1958 and 31 December 1996 were retrieved from the Database. Cases of second primary malignancies were extracted if the diagnosis date of the first and second malignancy differed by at least 1 month. Between 1958 and 1996, a total of 3498 patients developed a second primary malignancy among 53 159 HLPD patients.

### Statistical analysis

Person-years at risk for second malignancy were accumulated for each subject from the date of diagnosis of the first primary malignancy to that of a second primary malignancy, date of death, date of emigration, or December 31, 1996, whichever came first. Person-years were classified by sex, 5-year age group, 5-year calendar period (1958–1962, 1988–1992, 1993–1996), 2 types of residential area (3 counties of large cities vs other), 4 occupational groups (farmer, worker, professional and other), time since entry to the cohort (< 1, 1–9 and > 9 years) and family history of HLPD in parents and siblings. A cut-off point of 9 years after the first diagnosis was selected to explore the potential radiotherapy- and chemotherapy-induced risk for second primary malignancies (Travis et al, 1991, 1993; Swerdlow et al, 1997, 2000; Metayer et al, 2000).

Expected numbers were obtained by assuming that these persons experienced the same cancer incidence as prevailed in the corresponding general population in the Database and site-, sex-, age-, period-, residence- and occupation-specific rates were applied to the appropriate person-years at risk. Standardized incidence ratios (SIR) of a second cancer were taken to be the ratio of observed (O) to expected (E) numbers of second cancers. Confidence intervals (95%CI) for SIR were calculated assuming that the cases followed a Poisson distribution (Esteve et al, 1994).

### RESULTS

Table 1 reports the characteristics of HLPD patients by subgroup. Among 53 159 HLPD patients, with 264 190 person-years accumulated, 36% (18 960/53 159) were NHL, 10% (5353/53 159) HD, 16% (8656/53 159) myeloma, 15% (8098/53 159) lymphoid leukaemia, 13% (1727/53 159) myeloid leukaemia and 9% (4965/53 159) other leukaemia. Because other leukaemia is a mixed group, it will not be considered in further analyses. A total of 1.6% of HLPD (834/53 159) were familial. Familial lymphoma and leukaemia showed an at least 12 years earlier median age of onset than sporadic cases. Among 834 second primary malignancies, 51 (6.1%) had an HLPD-affected parent or sib, including 8 for second HLPD, 4 for melanoma or squamous cell carcinoma of the skin and 39 for solid tumours. Because of the small numbers, familial cases were not analysed separately.

Table 2 reports the overall risk of second primary cancer at specific sites after NHL and HD. For NHL patients, there were significant excesses of cancers of the tongue, small intestine, nose (2 of 6 cases were undifferentiated cancers), kidney, skin (squamous

**Table 1** Characteristics of patients with an initial HLPD[a]

| Characteristics | Type of initial HLPD: NHL | HD | Myeloma | Lymphoid leukaemia | Myeloid leukaemia | Other leukaemia | Total HLPD |
|---|---|---|---|---|---|---|---|
| Number of patients | 18 960 | 5353 | 8656 | 8098 | 7127 | 4965 | 53 159 |
| Male | 11 137 | 3258 | 5036 | 5118 | 3909 | 2769 | 31 227 |
| Female | 7823 | 2095 | 3620 | 2980 | 3218 | 2196 | 21 932 |
| Familial[b] | 300 | 131 | 93 | 123 | 110 | 77 | 834 |
| Sporadic | 18 660 | 5222 | 8563 | 7975 | 7017 | 4888 | 52 325 |
| Person-years at risk | 94 088 | 46 206 | 32 255 | 43 330 | 21 776 | 26 535 | 264 190 |
| Median age at 1st diagnosis (years) | 64 | 41 | 68 | 63 | 59 | 62 | 63 |
| Familial | 52 | 29 | 65 | 51 | 40 | 48 | 50 |
| Sporadic | 64 | 42 | 68 | 63 | 59 | 62 | 63 |
| Median length of follow-up (years) | 2.5 | 5.5 | 2.5 | 3.5 | 1.5 | 2.5 | 2.5 |
| Number of second primary cancers | 1354 | 411 | 475 | 659 | 170 | 429 | 3498 |
| Percent with second malignancy (%) | 7.1% | 7.7% | 5.5% | 8.1% | 2.4% | 8.6% | 6.6% |
| Median interval between 1st and 2nd diagnosis (years) | 4.4 | 9.3 | 2.9 | 3.8 | 1.9 | 6.2 | 6.5 |

[a]NHL: Non-Hodgkin's lymphoma (ICD-7 code: 200, 202); HD: Hodgkin's lymphoma (ICD-7 code: 201); multiple myeloma (ICD-7 code: 203); lymphoid leukaemia (ICD-7 code: 204); myeloid leukaemia (ICD-7 code: 205); other leukaemia (ICD-7 code: 206–209); HLPD: haematolymphopropoliferative disorder (ICD-7 code: 200–209). [b]Familial: patients had a HLPD-affected parent or sib.

© 2001 Cancer Research Campaign

**Table 2** Site-specific risk of second primary malignancy following HLPD[a]

| Second cancer site | Non-Hodgkin's lymphoma | | | Hodgkin's disease | | |
|---|---|---|---|---|---|---|
| (ICD-7 code) | O | SIR | (95% CI) | O | SIR | (95% CI) |
| Lip (140) | 8 | 1.29 | (0.55 – 2.56) | 1 | 0.72 | (0.00 – 4.11) |
| Tongue (141) | 9 | **3.03** | (**1.38** – **5.78**) | 1 | 1.38 | (0.00 – 7.90) |
| Salivary glands (142) | 5 | 2.10 | (0.66 – 4.94) | 5 | **8.00** | (**2.52** – **18.8**) |
| Mouth (143, 144) | 5 | 1.04 | (0.33 – 2.44) | 1 | 0.93 | (0.00 – 5.35) |
| Mesopharynx (145) | 2 | 1.06 | (0.10 – 3.88) | 0 | 0.00 | (0.00 – 8.12) |
| Nasopharynx (146) | 3 | 2.79 | (0.53 – 8.26) | 3 | **9.15** | (**1.73** – **27.1**) |
| Hypopharynx (147) | 1 | 0.51 | (0.00 – 2.94) | 0 | 0.00 | (0.00 – 9.22) |
| Other pharynx (148) | 0 | 0.00 | (0.00 – 30.6) | 1 | 31.57 | (0.01 – 181) |
| Oesophagus (150) | 10 | 1.00 | (0.48 – 1.85) | 3 | 1.54 | (0.29 – 4.55) |
| Stomach (151) | 57 | 1.12 | (0.85 – 1.45) | 18 | **1.69** | (**1.00** – **2.68**) |
| Small intestine (152) | 16 | **3.10** | (**1.77** – **5.05**) | **0** | 0.00 | (0.86 – 3.52) |
| Colon (153) | 73 | 0.82 | (0.64 – 1.03) | 29 | **1.72** | (**1.15** – **2.47**) |
| Rectum (154) | 39 | 0.75 | (0.53 – 1.02) | 1 | 1.09 | (0.54 – 1.95) |
| Anus (1541) | 5 | 2.38 | (0.75 – 5.60) | 1 | 2.30 | (0.00 – 13.2) |
| Liver (155, 156) | 42 | 1.13 | (0.82 – 1.53) | 7 | 1.01 | (0.40 – 2.10) |
| Pancreas (157) | 28 | 0.78 | (0.52 – 1.13) | 8 | 1.13 | (0.48 – 2.23) |
| Nose and nasal sinuses (160) | 6 | **3.25** | (**1.17** – **7.12**) | **0** | **0.00** | (**0.00** – 9.20) |
| Larynx (161) | 7 | 0.99 | (0.39 – 2.06) | 1 | 0.61 | (0.00 – 3.52) |
| Lung (162, 163) | 99 | 1.08 | (0.88 – 1.32) | 34 | **1.80** | (**1.24** – **2.51**) |
| Breast (170) | 73 | **0.71** | (**0.56** – **0.89**) | 45 | **1.73** | (**1.26** – **2.31**) |
| Cervix (171) | 9 | 0.90 | (0.41 –1.71) | 3 | 0.73 | (0.14 – 2.16) |
| Endometrium (172) | 12 | **0.51** | (**0.26** – **0.90**) | 3 | 0.59 | (0.11 – 1.75) |
| Other uteri (173, 174) | 3 | 1.26 | (0.24 – 3.74) | 0 | 0.00 | (0.00 – 6.05) |
| Ovary (175) | 13 | 0.64 | (0.34 – 1.09) | 7 | 1.31 | (0.52 – 2.72) |
| Other female genital organs (176) | 3 | 0.71 | (0.13 – 2.10) | 2 | 2.50 | (0.24 – 9.19) |
| Prostate (177) | 159 | **0.75** | (**0.64** – **0.88**) | 33 | 0.94 | (0.64 – 1.32) |
| Testis (178) | 5 | 2.58 | (0.82 – 6.08) | 0 | 0.00 | (0.00 – 2.20) |
| Other male genital organs (179) | 3 | 1.39 | (0.26 – 4.12) | 0 | 0.00 | (0.00 – 7.69) |
| Kidney (180) | 57 | **1.58** | (**1.20** – **2.05**) | 13 | 1.63 | (0.86 – 2.79) |
| Urinary bladder (181) | 70 | 1.09 | (0.85 – 1.37) | 10 | 0.80 | (0.38 – 1.49) |
| Melanoma of the skin (190) | 33 | 1.14 | (0.78 – 1.60) | 16 | **1.83** | (**1.04** – **2.98**) |
| SCC of the skin (191) | 167 | **3.54** | (**3.02** – **4.12**) | 23 | **2.89** | (**1.83** – **4.35**) |
| Eye (192) | 1 | 0.38 | (0.00 – 2.15) | 2 | 2.90 | (0.27 – 10.7) |
| Nervous system (193) | 40 | **1.45** | (**1.04** – **1.98**) | 15 | **1.81** | (**1.01** – **3.00**) |
| Thyroid (194) | 7 | 1.07 | (0.43 – 2.22) | 10 | **4.60** | (**2.19** – **8.49**) |
| Other endocrine glands (195) | 23 | 1.32 | (0.83 – 1.98) | 3 | 0.66 | (0.13 – 1.96) |
| Bone (196) | 1 | 0.77 | (0.00 – 4.39) | 1 | 1.92 | (0.00 – 11.0) |
| Soft tissues (197) | 7 | 1.03 | (0.41 – 2.14) | 7 | **3.98** | (**1.58** – **8.26**) |
| Non-Hodgkin's lymphoma (200, 202) | 79 | **2.29** | (**1.82** – **2.86**) | 41 | **5.53** | (**3.96** – **7.50**) |
| Hodgkin's disease (201) | 25 | **6.53** | (**4.22** – **9.66**) | **3** | 1.81 | (0.34 – 5.36) |
| Myeloma (203) | 16 | 0.88 | (0.50 – 1.44) | 4 | 1.13 | (0.29 – 2.93) |
| Lymphoid leukaemia (204) | 53 | **4.07** | (**3.05** – **5.33**) | 7 | **2.63** | (**1.04** – **5.44**) |
| Myeloid leukaemia (205) | 25 | **2.55** | (**1.65** – **3.77**) | 19 | **7.67** | (**4.61** – **12.0**) |
| Other leukaemia (206–209) | 9 | 1.23 | (0.56 – 2.35) | 9 | **5.67** | (**2.57** – **10.8**) |
| Other sites | 46 | 1.14 | (0.83 – 1.52 | 11 | 1.43 | (0.71 – 2.56) |
| All HLPD | 207 | **2.39** | (**2.08** – **2.74**) | 83 | **4.29** | (**3.42** – **5.32**) |
| All skin sites | 200 | **2.63** | (**2.28** – **3.02**) | 39 | **2.34** | (**1.66** – **3.20**) |
| All solid tissues | 947 | 0.96 | (0.90 – 1.02) | 289 | **1.40** | (**1.24** – **1.57**) |
| All sites | 1354 | **1.18** | (**1.12** – **1.24**) | 411 | **1.69** | (**1.53** – **1.86**) |

[a]SCC: squamous cell carcinoma; HLPD: haematolymphoproliferative disorder; O: observed cases; SIR: standardised incidence ratio; CI: confidence interval; Bold type: 95% CI did not include 1.00.

cell carcinoma, SCC), nervous system, NHL, HD and leukaemia, whereas there were significant decreased risks of cancers of the breast, endometrium and prostate. The SIR of 3.54 for SCC of skin was the highest among all solid cancers. For HD patients, significant excesses were seen for cancers of the salivary glands (5 cases, 2 of these undifferentiated), nasopharynx (3 cases, 2 undifferentiated), stomach (18 cases, all but one adenocarcinomas), colon, lung, breast, skin (both melanoma and SCC), nervous system, thyroid, soft tissues and leukaemias. Compared with the general population, NHL and HD patients had an excess of HLPD and skin tumours, but only HD patients were at increased risk for all second solid tumours (SIR = 1.40, 95% CI = 1.24–1.57).

Table 3 presents the risks of second malignancies following NHL by sex, age at first diagnosis and follow-up time at selected sites where there was a significant excess. Males and females had a similar risk pattern (increased or decreased) at all selected sites. Increased risks of second malignancy were observed over all age groups and follow-up times for small intestine, nose, skin SCC, NHL, HD and leukaemias. The SIRs for cancers of the tongue, small intestine and skin and for NHL, HD and LL were highest when the age at first diagnosis was under 40 years. An increased risk of breast cancer after NHL was confined to those whose age at first diagnosis was under 40 years, whereas those whose age at first diagnosis was ≥ 40 years had a significantly decreased risk

© 2001 Cancer Research Campaign 

**Table 3** Site-specific risk of second malignancy following non-Hodgkin's lymphoma by sex, age at first diagnosis and follow-up time[a]

| Second cancer site | Sex | | | | Age at first diagnosis (years) | | | | | | | | Year(s) after first diagnosis | | | | | |
|---|---|---|---|---|---|---|---|---|---|---|---|---|---|---|---|---|---|---|
| | Male | | Female | | <40 | | 40–54 | | 55–64 | | >64 | | <1 | | 1–9 | | >9 | |
| | O | SIR | O | SIR | O | SIR | O | SIR | O | SIR | O | SIR | O | SIR | O | SIR | O | SIR |
| Tongue | 7 | **3.48** | 2 | 2.08 | 2 | **15.09** | 2 | 3.09 | **4** | **5.09** | 1 | 0.71 | 0 | 0.00 | 7 | **3.54** | 2 | 2.93 |
| Small Intestine | **12** | **3.67** | 4 | 2.11 | 2 | **12.11** | 3 | 3.22 | 2 | 1.53 | 9 | **3.27** | 2 | 3.66 | 12 | **3.50** | 2 | 1.68 |
| Nose and nasal sinuses | 4 | 3.10 | 2 | 3.57 | 1 | 16.34 | 1 | 2.80 | 2 | 4.19 | 2 | 2.10 | 1 | 4.91 | 3 | 2.43 | 2 | 4.91 |
| Kidney | **42** | **1.77** | 15 | 1.23 | 0 | 0.00 | 7 | 1.02 | 14 | 1.39 | **36** | **2.00** | 17 | **4.38** | 32 | 1.33 | 8 | 1.00 |
| SCC of the skin | 127 | **3.82** | 40 | **2.86** | 4 | **6.01** | 9 | 1.64 | 40 | **4.64** | 114 | **3.52** | 8 | 1.61 | 124 | **4.01** | 35 | **3.10** |
| Nervous system | 21 | 1.37 | 19 | 1.56 | 6 | 2.74 | 8 | 1.17 | 16 | **2.05** | 10 | 0.93 | 4 | 1.40 | 26 | 1.41 | 10 | 1.61 |
| Non-Hodgkin's lymphoma | 52 | **2.32** | 27 | **2.24** | 9 | **6.82** | 26 | **4.18** | 18 | **2.12** | 26 | 1.41 | 6 | 1.60 | 54 | **2.37** | 19 | **2.42** |
| Hodgkin's disease | 17 | **6.70** | 8 | **6.19** | 7 | **14.20** | 7 | **8.78** | 5 | **5.36** | 6 | **3.74** | 1 | 2.34 | 16 | **6.29** | 8 | **9.34** |
| Lymphoid leukaemia | 37 | **4.09** | 16 | **4.01** | 5 | **12.29** | 14 | **6.80** | 23 | **7.17** | 11 | 1.50 | 6 | **4.26** | 35 | **4.04** | 12 | **4.05** |
| Myeloid leukaemia | 13 | **2.15** | 12 | **3.20** | 1 | 2.03 | 5 | 2.80 | 5 | **2.09** | 14 | **2.73** | 1 | 0.96 | 19 | **2.92** | 5 | 2.23 |
| All HLPD | 135 | **2.41** | 72 | **2.36** | 23 | **7.01** | 58 | **3.90** | 59 | **2.78** | 67 | **1.42** | 16 | 1.70 | 142 | **2.47** | 49 | **2.48** |
| All skin sites | 152 | **2.97** | 48 | **1.92** | 7 | 2.30 | 13 | 1.04 | 48 | **2.97** | 132 | **2.98** | 11 | 1.41 | 148 | **2.94** | 41 | **2.28** |
| All sites | 885 | **1.22** | 469 | **1.11** | 84 | **2.45** | 239 | **1.22** | 354 | **1.25** | 677 | 1.07 | 147 | **1.20** | 875 | **1.15** | 332 | **1.26** |

[a]SCC: squamous cell carcinoma; HLPD: haematolymphoproliferative disorder; O: observed cases; SIR: standardised incidence ratio; Bold type: 95% CI did not include 1.00.

*© 2001 Cancer Research Campaign*

**Table 4** Site-specific risk of second malignancy following Hodgkin's disease by sex, age at first diagnosis and follow-up time[a]

| Second cancer site | Sex | | | | Age at first diagnosis (years) | | | | | | | | Year(s) after first diagnosis | | | | | |
|---|---|---|---|---|---|---|---|---|---|---|---|---|---|---|---|---|---|---|
| | Male | | Female | | < 40 | | 40–54 | | 55–64 | | > 64 | | < 1 | | 1–9 | | > 9 | |
| | O | SIR | O | SIR | O | SIR | O | SIR | O | SIR | O | SIR | O | SIR | O | SIR | O | SIR |
| Salivary glands | 3 | **7.82** | 2 | 8.26 | 3 | **15.36** | 1 | 5.15 | 1 | 8.34 | 0 | 0.00 | 0 | 0.00 | 2 | 6.80 | 3 | **10.5** |
| Nasopharynx | 3 | **11.8** | 0 | 0.00 | 1 | 10.83 | 2 | **17.14** | 0 | 0.00 | 0 | 0.00 | 0 | 0.00 | 2 | **12.22** | 1 | 7.15 |
| Stomach | 15 | **1.92** | 3 | 1.06 | 4 | 3.43 | 9 | **2.71** | 1 | 0.36 | 4 | 1.19 | 0 | 0.00 | 6 | 1.22 | 12 | **2.53** |
| Colon | 22 | **2.17** | 7 | 1.04 | 6 | 2.46 | 8 | 1.52 | 6 | 1.48 | 9 | 1.77 | 0 | 0.00 | 15 | **2.04** | 14 | 1.70 |
| Lung | 25 | **1.67** | 9 | **2.29** | 8 | **2.68** | 10 | 1.50 | 13 | **2.77** | 3 | 0.66 | 2 | 1.49 | 16 | **1.94** | 16 | 1.72 |
| Breast | 0 | **0.00** | 45 | **1.74** | 31 | **3.36** | 4 | 0.50 | 3 | 0.64 | 7 | 1.69 | 0 | 0.00 | 11 | 1.00 | 34 | **2.51** |
| Melanoma of the skin | 12 | **2.33** | 4 | 1.11 | 7 | 1.76 | 3 | 1.19 | 5 | **4.19** | 1 | 0.96 | 3 | **6.65** | 9 | **2.29** | 4 | 0.92 |
| SCC of the skin | 15 | **2.63** | 8 | **3.55** | 4 | **4.22** | 4 | 1.73 | 7 | **3.77** | 8 | **2.83** | 0 | 0.00 | 9 | **2.88** | 14 | **3.30** |
| Nervous system | 9 | 1.86 | 6 | 1.75 | 7 | 2.12 | 3 | 1.18 | 2 | 1.43 | 3 | 2.89 | 2 | 3.85 | 7 | 1.77 | 6 | 1.58 |
| Thyroid | 3 | 3.73 | 7 | **5.11** | 7 | **7.02** | 0 | 0.00 | 3 | **9.65** | 0 | 0.00 | 1 | 7.21 | 3 | 2.78 | 6 | **6.28** |
| Soft tissues | 3 | 2.70 | 4 | **6.20** | 3 | 5.13 | 3 | **5.81** | 0 | 0.00 | 1 | 2.99 | 0 | 0.00 | 3 | 3.61 | 4 | **4.96** |
| Non-Hodgkin's lymphoma | 29 | **5.69** | 12 | **5.15** | 16 | **8.42** | 7 | **2.96** | 3 | 2.02 | 15 | **9.00** | 2 | 4.18 | 23 | **7.18** | 16 | **4.28** |
| Lymphoid leukaemia | 5 | 2.58 | 2 | 2.73 | 2 | 4.18 | 2 | 2.39 | 3 | 4.74 | 0 | 0.00 | 0 | 0.00 | 5 | **4.19** | 2 | 1.58 |
| Myeloid leukaemia | 13 | **8.25** | 6 | **6.64** | 10 | **13.06** | 5 | **6.77** | 3 | **6.45** | 1 | 1.97 | 0 | 0.00 | 15 | **12.88** | 4 | 3.51 |
| All HLPD | 58 | **4.42** | 25 | **4.01** | 38 | **7.99** | 17 | **2.85** | 11 | **2.72** | 17 | **3.72** | 3 | 2.14 | 54 | **6.16** | 26 | **2.84** |
| All skin sites | 27 | **2.49** | 12 | **2.05** | 11 | **2.23** | 7 | 1.45 | 12 | **3.93** | 9 | **2.33** | 3 | 2.92 | 18 | **2.55** | 18 | **2.09** |
| All solid tissues | 167 | **1.35** | 122 | **1.47** | 97 | **2.33** | 80 | 1.21 | 50 | 1.07 | 62 | 1.18 | 11 | 0.75 | 124 | **1.37** | 154 | **1.52** |
| All sites | 252 | **1.70** | 159 | **1.68** | 146 | **2.85** | 104 | **1.35** | 73 | **1.36** | 88 | **1.44** | 17 | 0.99 | 196 | **1.84** | 198 | **1.66** |

[a]SCC: squamous cell carcinoma; HLPD: haematolymphoproliferative disorder; O: observed cases; SIR: standardised incidence ratio; bold type: 95% CI did not include 1.00.

© 2001 Cancer Research Campaign

**Table 5** Site-specific risk of second malignancy following multiple myeloma and leukaemia by follow-up time and sex[a]

| First cancer | Second cancer site | Year(s) after first diagnosis | | | | | | Total | | |
|---|---|---|---|---|---|---|---|---|---|---|
| | | < 1 | | 1–9 | | > 9 | | | | |
| | | O | SIR | O | SIR | O | SIR | O | SIR | (95% CI) |
| Myeloma | SCC of the skin | 6 | 2.15 | 20 | 1.29 | 3 | 0.97 | 29 | 1.36 | (0.91–1.95) |
| | Nervous system | 4 | 2.68 | 11 | 1.36 | 0 | 0.00 | 15 | 1.39 | (0.77–2.30) |
| | Non-Hodgkin's lymphoma | 4 | 2.08 | 19 | **1.78** | 2 | 1.11 | 25 | **1.74** | (**1.12–2.57**) |
| | Hodgkin's disease | 0 | 0.00 | 2 | 1.70 | 0 | 0.00 | 2 | 1.25 | (0.12–4.59) |
| | Myeloma | 0 | 0.00 | 3 | 0.48 | 2 | 1.89 | 5 | 0.59 | (0.19–1.38) |
| | Lymphoid leukaemia | 0 | 0.00 | 2 | 0.46 | **1** | 1.33 | 3 | 0.51 | (0.10–1.51) |
| | Myeloid leukaemia | 1 | 1.72 | 30 | **9.50** | 4 | **7.43** | 35 | **8.19** | (**5.70–11.4**) |
| | All HLPD | 5 | 0.96 | 67 | **2.40** | 11 | **2.31** | 83 | **2.19** | (**1.74–2.71**) |
| | All skin sites | 6 | 1.42 | 29 | 1.23 | 4 | 0.92 | 39 | 1.22 | (0.86–1.66) |
| | All solid tissues | 64 | 1.08 | 248 | **0.77** | 41 | **0.73** | 353 | **0.81** | (**0.73–0.90**) |
| | All sites | 75 | 1.09 | 344 | 0.92 | 56 | 0.86 | 475 | 0.94 | (0.86–1.03) |
| Lymphoid leukaemia | SCC of the skin | 2 | 0.96 | 98 | **6.14** | 21 | **4.33** | 121 | **5.28** | (**4.38–6.31**) |
| | Nervous system | 7 | **6.09** | 9 | 1.08 | 6 | **3.10** | 22 | **1.93** | (**1.21–2.92**) |
| | Non-Hodgkin's lymphoma | 0 | 0.00 | 47 | **4.33** | 11 | **3.94** | 58 | **3.85** | (**2.92–4.98**) |
| | Hodgkin's disease | 0 | 0.00 | 6 | **4.60** | 2 | 5.39 | 8 | **4.29** | (**1.83–8.50**) |
| | Myeloma | 0 | 0.00 | 2 | 0.32 | 1 | 0.64 | 3 | 0.35 | (0.07–1.04) |
| | Lymphoid leukaemia | 1 | 1.34 | 13 | **2.67** | 3 | 2.55 | 17 | **2.51** | (**1.46–4.02**) |
| | Myeloid leukaemia | 1 | 2.27 | 10 | **3.13** | 0 | 0.00 | 11 | **2.46** | (**1.22–4.42**) |
| | All HLPD | 2 | 0.51 | 81 | **2.80** | 18 | **2.44** | 101 | **2.51** | (**2.05–3.05**) |
| | All skin sites | 4 | 1.28 | 112 | **4.67** | 22 | **3.18** | 138 | **4.06** | (**3.41–4.79**) |
| | All solid tissues | 58 | **1.33** | 299 | 0.92 | 63 | **0.75** | 420 | 0.93 | (0.84–1.02) |
| | All sites | 64 | 1.26 | 492 | **1.30** | 103 | 1.04 | 659 | **1.25** | (**1.16–1.35**) |
| Myeloid leukaemia | SCC of the skin | 1 | 0.68 | 12 | **3.44** | 0 | 0.00 | 13 | 1.66 | (0.88–2.85) |
| | Nervous system | 1 | 1.04 | 8 | 2.62 | 1 | 0.73 | 10 | 1.86 | (0.88–3.43) |
| | Non-Hodgkin's lymphoma | 0 | 0.00 | 4 | 1.34 | 0 | 0.00 | 4 | 0.69 | (0.18–1.79) |
| | Hodgkin's disease | 0 | 0.00 | 0 | 0.00 | 0 | 0.00 | 0 | 0.00 | (0.00–4.51) |
| | Myeloma | 0 | 0.00 | 3 | 1.89 | 0 | 0.00 | 3 | 0.93 | (0.18–2.77) |
| | Lymphoid leukaemia | 0 | 0.00 | 4 | 3.41 | 1 | 1.38 | 5 | 2.13 | (0.67–5.01) |
| | Myeloid leukaemia | 2 | 4.86 | 13 | **12.8** | 2 | 3.80 | 17 | **8.70** | (**5.06–14.0**) |
| | All HLPD | 2 | 0.68 | 30 | **3.78** | 3 | 0.65 | 35 | **2.25** | (**1.57–3.14**) |
| | All skin sites | 1 | 0.42 | 14 | **2.18** | 0 | **0.00** | 15 | 1.15 | (0.64–1.90) |
| | All solid tissues | 29 | 0.87 | 76 | 0.84 | 15 | 0.28 | 120 | **0.68** | (**0.56–0.81**) |
| | All sites | 32 | 0.83 | 120 | 1.15 | 18 | **0.29** | 170 | **0.83** | (**0.71–0.96**) |

[a]SCC: squamous cell carcinoma; HLPD: haematolymphoproliferative disorder; O: observed cases; SIR: standardised incidence ratio; CI: confidence interval: Bold type: 95% CI did not include 1.00.

(data not shown). A significant increase in cancer of the kidney was confined to the first year of follow-up and this may be due to increased surveillance or chance. An excess of bladder cancer was confined to the period > 9 years of follow-up (data not shown).

Table 4 reports the risks of second malignancies following HD by sex, age at first diagnosis and follow-up time at selected sites where there was a significant excess. Males and females showed a similar risk pattern (increased or decreased) for cancers of salivary glands, lung, skin SCC, nervous system, thyroid and soft tissue and for haematolymphoproliferative tissues except multiple myeloma; by contrast, an increased risk of cancer of the nasopharynx, stomach, colon and melanoma skin was confined to male HD patients. An excess of female breast cancer was confined to patients whose initial diagnosis age was under 40 years and the period > 9 years of follow-up. Increased risks of cancers of colon, skin and nervous system, NHL and leukaemia were observed over all age groups and follow-up periods over 1 year. Increased risks of cancers of salivary glands, nasopharynx, stomach, lung, thyroid and soft tissues were mainly found in patients whose age at first diagnosis was under 55 years and ≤ 1 year since HD diagnosis. An increased risk of melanoma was seen in those under 65 years at first diagnosis and follow up period ≤ 9 years, with the trend decreasing from initial diagnosis.

Table 5 reports risks following myeloma and leukaemia by subgroup and follow-up time for second malignancies of skin, nervous system, haematolymphoproliferative tissues and all solid tissues. After myeloma, a significant increase was seen for SCC of the skin in males but not in both sexes; for NHL the overall SIR was increased. After lymphoid leukaemia, a significant increase in SIR was seen for SCC of the skin and NHL during period ≥1 year of follow-up and overall, for cancer of the nervous system during period <1 and > 9 years of follow-up and overall, for HD, lymphoid and myeloid leukaemia during 1–9 years of follow-up and overall. After myeloid leukaemia, a significantly increased risk was found only for recurrence. No excess of melanoma was observed (data not shown). The SIR for melanoma was highest after lymphoid leukaemia, 1.53 ($n$ = 17, 95% CI, 0.89–2.43); 15 of the cases were men, SIR 2.02 (95%CI, 1.13–3.34).

### DISCUSSION

The Swedish Family-Cancer Database was used in the present study and 6.1% of second HLPD cases had a family history of HLPD, which was higher than that for first HLPD (1.6%). The patients with a family history presented with their first HLPD on average 12 years earlier than patients with a sporadic disease. Our results are based on one of the largest cohorts of patients

*© 2001 Cancer Research Campaign*

with HLPD studied to date (totalling 53 159 patients, 264 190 person-years), enabling us to analyse site-specific risk of second cancer following HLPD. The large dataset permits description of age and latency patterns for second cancer risk at a broad spectrum of anatomic sites. In addition, the estimation of SIRs was based on adjustment for period, residence and socio-economic status, which may be potential confounding factors and mainly unadjusted in previous studies. Period is important, particularly for NHL, which has increased in incidence throughout the follow-up period, and even for other HLPDs because of changes in treatment. Although our data lack information about treatment, we divided the follow-up time into 3 periods, <1, 1–9 and > 9 years. The period > 9 years of follow-up may reveal the effects due to radiotherapy and chemotherapy at most solid tumours but leukaemias appear earlier (Travis et al, 1991, 1993; Swerdlow et al, 1997, 2000; Metayer et al, 2000); the first year of diagnosis may include recurrences and reveal the effects of medical surveillance. We consider the effect of follow-up in subsequent discussion.

HLPDs are a number of heterogeneous but related conditions. However, misclassification, particularly NHL to HD, may have taken place in the past (Travis et al, 1991; Abrahamsen et al, 1997; Kumar et al, 1997). The distinction between the related conditions, NHL and chronic LL, may also have been unclear in the past (Adami et al, 1995). The classification of HLPDs has undergone many changes during the past decades and whether these changes have affected the broad categories used in the present study remains unknown.

Treatment of HLPDs has undergone major changes during the period of the present study, 1958–96. Chemotherapy and radiotherapy have been used throughout the period but new antineoplastic agents and more aggressive therapies have been introduced (Adami et al, 1995). For some HLPDs, prognosis has improved markedly and, for example, the 5-year survival from HD has improved 3-fold in Sweden between 1962 and 1988 (Stenbeck and Rosen, 1995). The respective improvements for NHL, multiple myeloma and leukaemias have been about 1.5-fold. Radiotherapy and many types of chemotherapy carry a risk for second cancers and treatment of HD is known to increase the risks of acute leukaemia, NHL and of lung, gastro-intestinal, breast, thyroid, bone and connective tissue cancer (Kumar et al, 1997; Metayer et al, 2000; Swerdlow et al, 2000).

### NHL, HD, leukaemia and myeloma

Many studies have been published on second cancers after NHL. Common findings have been increases in acute leukaemia, cancers at lung, bladder, soft tissues, thyroid and kidney, melanoma and HD (Travis et al, 1991, 1993; Brennan et al, 2000). Excesses in melanoma and, particularly, skin SCC have been observed after NHL and chronic lymphoid leukemia (Adami et al, 1995; Levi et al, 1996). The present study, only second to the Adami study in accumulated person-years, noted increases in tongue, small intestinal, nasal, bladder, skin (SCC but not melanoma), and nervous system cancers, in addition to all types of HLPD. Many of these increases in earlier studies, have been ascribed to the effects of treatment, though other possible causes have probably been overlooked (Travis et al, 1991). In contrast, we found no increases in gastro-intestinal (except for small intestine) and lung cancers nor, notably, in melanoma. On the other hand, details of nasal cancer (our SIR 3.25) have been reported in only one study and no increase was noted (Brennan et al, 2000). We have recently drawn attention to the occurrence of nasal cancer as a second cancer after skin SCC and speculated on the possible aetiological role of EBV, perhaps in immunocompromised individuals (Hemminki and Dong, 2000b, 2000c). We have recently noted an excess of second NHL after nasal cancer (Dong and Hemminki, 2001b). These epidemiological findings may not be fortuitous. Another site, which was increased after NHL, was the nervous system; this site was also in excess after lymphoid leukaemia and the association is thus unlikely to be fortuitous. An increase has also been noted in a previous study (SIR 2.33) and in another one for males only (Travis et al, 1991, 1993). Treatment effects in nasal and nervous system tumours were not suggested when the risks were analysed according to the follow-up time (Table 3).

NHL and HD were increased after each other, with high SIRs of 5.5–6.5 (Table 2). Both have been treated with aggressive therapy. Yet the spectrum of second malignancies observed was quite different: among solid tumours only skin SCC and nervous system cancers were increased after both NHL and HD. One reason for the different effects may be in the age distribution; the median age of the first HD diagnosis was 41 years compared to 64 for NHL. There may also be other biological explanations. Many large follow-up studies have been carried out after HD with the aim of quantifying carcinogenic risks of radiation and chemotherapy (Swerdlow et al, 1997, 2000; Metayer et al, 2000). All the sites that were increased in the present study were also noted in these previous studies, including salivary glands and upper aerodigestive tract (without further subdivision), but with the notable exception of skin cancer. The SIR for nonmelanoma skin cancer was 1.4 in the English study and it was excluded from further analysis because registration was assumed to be seriously incomplete (Swerdlow et al, 2000). Skin cancer and melanoma are discussed below.

It is of interest that nasopharynx (SIR 9.15) and stomach (SIR 1.69) together with HD and certain types of NHL are considered as potentially EBV-related (IARC, 1997). The increases at salivary glands and nasopharynx were the highest among second malignancies after HD. Histology showed that more than expected of these tumours were undifferentiated cancers though the number of cases was small. Analysis by follow-up time (Table 4) showed increases in SIR towards the end of follow-up, suggesting either treatment related effects or possibly slow malignant viral transformation.

Myeloma was followed by an excess of NHL and leukaemia. Lymphoid leukaemia was followed by excesses of skin SCC (SIR 5.28), nervous system cancer (1.93), and many types of HLPD. The distribution of skin and nervous system cancers after diagnosis of lymphoid leukaemia gave no suggestion of being treatment-related.

### Melanoma and SCC of skin following HLPD

UVR is a common risk factor for melanoma and skin SCC. For skin SCC, the total life-time UVR-dose is considered important; for melanoma high intermittent exposures in childhood and adolescence are considered important (Adami et al, 1995; English et al, 1997). A second aetiological factor is defective immune function and skin SCC and NHL are greatly increased in organ transplant patients (Kinlen, 1992; IARC, 1997). The third aetiological factor could be chemo- and radiotherapy, radiation being known to increase the risk of skin SCC though data on chemotherapy are not consistent (IARC, 1990; Metayer et al, 2000; Swerdlow et al, 2000).

*© 2001 Cancer Research Campaign* 

An excess of melanoma after NHL and chronic lymphoid leukaemia has been observed in most previous studies (Travis et al, 1991, 1993; Adami et al, 1995; Levi et al, 1996; Brennan et al, 2000), whereas our data were negative for NHL and only marginally positive for lymphoid leukaemia. After HD, melanoma was increased but the highest SIR was found immediately after the initial diagnosis, suggesting surveillance bias. In most of the previous studies, cited above, the risks for melanoma were also high immediately after the initial diagnosis. Taken together, the data suggest a modest increase of melanoma risk after NHL and chronic lymphoid leukaemia.

The case for skin SCC is much stronger. In an earlier study the risks after both NHL and chronic lymphoid leukaemia were more than 2 times higher for skin SCC than melanoma (Adami et al, 1995). Excesses of NHL and chronic lymphoid leukaemia have been noted after skin SCC (Adami et al, 1995; Levi et al, 1996). An increase in all lymphomas, 80% of which were NHL, has been observed even after in situ SCC of skin, a benign tumour for which the primary treatment has been surgery only (Hemminki and Dong, 2000c). The present data were convincing showing high SIRs for skin SCC after NHL, HD and lymphoid leukaemia. The excess of skin SCC after HD, only previously documented for non-melanoma skin cancer (Swerdlow et al, 1997) was robust ($n$ = 23, SIR 2.89, 95%CI, 1.83–4.35). There was no evidence that the excess of skin SCC as a second malignancy was due to surveillance bias or therapy.

### Conclusions

There has been much interest in the aetiology of NHL, melanoma and skin SCC because they have increased in most Western countries, in Sweden more than any other cancer (Centre for Epidemiology, 1999). Our data unify the fragmentary earlier evidence to point to the aetiological links between NHL, HD, chronic lymphoid leukaemia and skin SCC. The common mechanism may be a (possibly) transient impairment of immunological surveillance that allows escape of tumours cells and formation of SCC. Depressed immune function may also be a contributing cause for the initial NHL, HD and lymphoid leukaemia, and the long-term chemo- and radiotherapy may increase such depression. This interpretation will not explain the worldwide temporal increase in NHL alone among HLPDs (Cartwright et al, 1994). However, data on the temporal trends on subtypes of leukaemias are limited and those on HD show complex patterns of increase among young and large decrease among old patients (Abrahamsen et al, 1997; Chen et al, 1997). UVR-induced immunosuppression has been suggested to contribute to the increasing trends in both NHL and melanoma, or, as consistent with our results, in NHL and skin BCC (Cartwright et al, 1994; Adami et al, 1995; Levi et al, 1996; Brennan et al, 2000). There is evidence from epidemiological studies on melanoma that use of sunscreens is a risk factor for melanoma (IARC, 2001). Among the 15 case–control studies on melanoma reviewed by the IARC working group, 8 showed significantly higher risks among sunscreen users. Although some of these studies may have problems with confounding, the increasing use of sunscreens deserves further research. Allergies, asthma and atopy are other immune-related groups of diseases that have been increasing in prevalence in the developed world. In these conditions the immune system is hyperactive, and there is evidence for allergies protecting against NHL (Volkers, 1999).

We observed associations to putative EBV-related sites, salivary glands, nasopharynx and stomach after HD; nasal cancer was increased after NHL but the link to EBV remains to be established. Undifferentiated cancers were in excess at sites other than the stomach and the number of cases was too small to allow conclusions. Nervous system cancer was the only solid tumour, together with skin SCC, that was increased after NHL, HD and lymphoid leukaemia.

### ACKNOWLEDGEMENTS

The study was supported by the Swedish Cancer Society.

### REFERENCES

Abrahamsen F, Egeland T, Hansen S, Langholm R, Holte H and Kvaloy S (1997) Hodgkin's disease in a national and hospital population: trends over 20 years. *Eur J Cancer* **33**: 2380–2383

Adami J, Frisch M, Yuen J, Glimelius B and Melbye M (1995) Evidence of an association between non-Hodgkin's lymphoma and skin cancer. *Br Med J* **310**: 1491–1495

Brennan P, Coates M, Armstrong B, Colin D and Boffetta P (2000) Second primary neoplasms folloging non-Hodgkin's lymphoma in New South Wales, Australia. *Br J Cancer* **82**: 1344–1349

Capalbo S, Trerotoli P, Ciancio C, Battista C, Serio G and Liso V (2000) Increased risk of lymphoproliferative disorders in relatives of patients with B-cell chronic lymphocytic leukemia: relevance of the degree of familial linkage. *Eur J Haematol* **65**: 114–117

Cartwright R, McNally R and Staines A (1994) The increasing incidence of non-Hodgkin's lymphoma (NHL): the possible role of sunlight. *Leuk Lymph* **14**: 387–394

Centre for Epidemiology (1999) Cancer Incidence in Sweden 1997 pp 1–114: Stockholm

Chen Y, Zheng T, Chou M, Boyle P and Holford T (1997) The increase of Hodgkin's disease incidence among young adults. Experience in Connecticut, 1935–1992. *Cancer* **79**: 2209–2218

Dong C and Hemminki K (2001a) Multiple primary cancers at colon, breast and skin (melanoma) as models for polygenic cancers. *Int J Cancer* **92**: 883–887

Dong C and Hemminki K (2001b) Risk of multiple primary cancers in nasal cancer patients. *Epidemiology* **12**: 367–369

English D, Armstrong B, Kricker A and Fleming C (1997) Sunlight and cancer. *Cancer Causes Control* **8**: 271–283

Esteve J, Benhamou E and Raymond L (1994) *Statistical Methods in Cancer Research*. Vol. 128. IARC Scientific Publication. IARC: Lyon

Greene M and Wilson J (1985) Second cancer following lymphatic and hematopoietic cancers in Connecticut, 1935–82. *NCI Monogr* **68**: 191–217

Groves F, Linet M, Travis L and Devesa S (2000) Cancer surveillance series: non-Hodgkin's lymphoma incidence by histologic subtype in the United States from 1978 through 1995. *J Natl Cancer Inst* **92**: 1240–1251

Hemminki K and Dong C (2000a) Familial relationships in squamous cell carcinoma of the skin. *Epidemiology* **11**: 309–314

Hemminki K and Dong C (2000b) Primary cancers following squamous cell carcinoma of the skin suggest involvement of Epstein-Barr virus. Letter to editor. *Epidemiology* **11**: 94

Hemminki K and Dong C (2000c) Subsequent cancers after in situ and invasive squamous cell carcinoma of the skin. *Arch Dermatol* **136**: 647–651

Hemminki K and Vaittinen P (1999) Familial cancers in a nation-wide family-cancer database: age distribution and prevalence. *Eur J Cancer* **35**: 1109–1111

Hemminki K, Vaittinen P and Kyyrönen P (1998) Age-specific familial risks in common cancers of the offspring. *Int J Cancer* **78**: 172–175

Horwitz M (1997) The genetics of familial leukemia. *Leukemia* **11**: 1347–1359

IARC (1990) *Cancer: Causes, Occurence and Control* Vol. 100. IARC Sci Publications. IARC: Lyon

IARC (1996) *IARC Monographs on the Evaluation of Carcinogenic Risks to Humans*. Vol. 67. Human immunodeficiency viruses and human T-cell lymphotrophic viruses. IARC: Lyon

IARC (1997) *IARC Monographs on the Evaluation of Carcinogenic Risks to Humans. Epstein-Barr virus and Kaposi's sarcoma. Herpesvirus/human herpesvirus 8* Vol. 70. IARC: Lyon

 *© 2001 Cancer Research Campaign*

IARC (2001) *Sunscreens* Vol. Vol. 5. IARC Handbooks of Cancer Prevention. IARC: Lyon

Kinlen L (1992) Immunosuppression and cancer. In *Mechanisms of Carcinogenesis in Risk Identification*, Vainio H, Magee P, McGregor D and McMichael A (eds), Vol. IARC Sci Publ vol. 116. pp. 237–253. IARC: Lyon

Kumar V, Cotran R and Robbins S (1997) *Basic Pathology* W.B. Saunders: Philadelphia

Levi F, Randimbison L, Te V and La Vecchia C (1996) Non-Hodgkin's lymphoma, chronic lymphocytic leukaemias and skin cancers. *Br J Cancer* **74**: 1847–1850

Metayer C, Lynch C, Clarke E, Glimelius B, Storm H, Pukkala E, Joensuu T, van Leeuwen F, van't Veer M, Curtis R, Holowaty E, Andersson M, Wiklund T, Gospodarowicz M and Travis L (2000) Second cancers among long-term survivors of Hodgkin's disease diagnosed in childhood and adolescence. *J Clin Oncol* **18**: 2435–2443

Sankila R, Pukkala E and Teppo L (1995) Risk of subsequent malignant neoplasms among 470000 cancer patients in Finland 1953–1991. *Int J Cancer* **60**: 464–470

Shugart Y, Hemminki K, Vaittinen P, Kingman A and Dong C (2000) A genetic study of Hodgkin's lymphoma: an estimate of heritability and anticipation based on the familial cancer database in Sweden. *Hum Genet* **106**: 553–556

Stenbeck M and Rosen M (1995) Cancer survival in Swedenin 1961–1991. *Acta Oncol* **34,** (Suppl 4), 1–124

Storm H and Prener A (1985) Second cancer following lymphatic and hematopoietic cancers in Denmark, 1943–1980. *NCI Monogr* **68**: 389–410

Swerdlow A, Barber J, Horwich A, Cunningham D, Milan S and Omar R (1997) Second malignancy in patients with Hodgkin's disease treated at the Royal Marsden Hospital. *Br J Cancer* **75**: 116–123

Swerdlow A, Barber J, Hudson G, Cunningham D, Gupta R, Hancock B, Horwich A, Lister T and Linch D (2000) Risk of second malignancy after Hodgkin's disease in a collaborative British cohort: the relation to age at treatment. *J Clin Oncol* **18**: 498–509

Travis L, Curtis R, Boice JJ, Hankey B and Fraumeni JJ (1991) Second cancers following non-Hodgkin's lymphoma. *Cancer* **67**: 2002–2009

Travis L, Curtis R, Glimelius B, Holowaty E, Van Leeuwen F, Lynch C, Adami J, Gospodarowics M, Wacholder S and Inskip P (1993) Second cancers among long-term survivors of non-Hodgkin's lymphoma. *J Natl Cancer Inst* **85**: 1932–1937

Vaittinen P and Hemminki K (2000) Risk factors and age-incidence relationships for contralateral breast cancer. *Int J Cancer* **88**: 998–1002

Volkers N (1999) Wheezing, sneezing, and cancer risk – still an open door. *J Natl Cancer Inst* **91**: 1916–1918

Wiernik P, Wang S, Hu X.-P, Marino P and Paietta E (2000) Age of onset evidence for anticipation in familial non-Hodgkin's lymphoma. *Br J Haematol* **108**: 72–79

Yuille M, Houlston R and Catovsky D (1998) Anticipation in familial chronic lymphocytic leukaemia. *Leukemia* **12**: 1696–1698

*© 2001 Cancer Research Campaign*
